# Prognostic relevance of hemodialysis for short-term survival in patients after LVAD implantation

**DOI:** 10.1038/s41598-018-26515-0

**Published:** 2018-06-04

**Authors:** Bastian Schmack, Leonie Grossekettler, Alexander Weymann, Joel Schamroth, Anton Sabashnikov, Philip W. Raake, Aron F. Popov, Ashham Mansur, Matthias Karck, Vedat Schwenger, Arjang Ruhparwar

**Affiliations:** 1 Department of Cardiac Surgery, University Hospital of Heidelberg, Heidelberg, Germany; 2 Department of Internal Medicine III, Cardiology, Angiology and Pneumology, University Hospital of Heidelberg, Heidelberg, Germany; 3 Department of Cardiac Surgery, University Hospital Oldenburg, Oldenburg, Germany; 4 Department of Cardiothoracic Transplantation and Mechanical Circulatory Support, Royal Brompton and Harefield NHS Foundation Trust, Harefield, UK; 5 Department of Cardiothoracic Surgery, University Hospital of Cologne, Cologne, Germany; 6 Department of Thoracic and Cardiovascular Surgery, Johann-Wolfgang-Goethe University, Frankfurt am Main, Germany; 7 University Medical Center, University of Goettingen, Department of Anesthesiology, Emergency and Intensive Care Medicine, Goettingen, Germany; 8 Department of Internal Medicine I, Section Nephrology, University Hospital of Heidelberg, Heidelberg, Germany

## Abstract

End-stage heart failure (HF) is associated with renal failure (RF). This study aimed to determine the prognostic influence of RF and post-operative hemodialysis on short-term survival following left ventricular assist device (LVAD) implantation. This retrospective study includes 68 patients undergoing LVAD treatment. Kidney function was recorded prior to LVAD implantation, immediately afterwards and after 30 days, noting the need for hemodialysis. Median pre-operative Interagency Registry for Mechanically Assisted Circulatory Support (INTERMACS) classification was 3.47 ± 1.08. 30 days after implantation there was a significant improvement of estimated glomerular filtration rate (eGFR) and reduction of blood urea nitrogen (BUN). Of pre-operative RF parameters, BUN was associated with increased mortality and need for early post-operative hemodialysis. Post-operative hemodialysis was associated with significantly lower short-term survival, while pre-operative hemodialysis did not impact mortality. Post-operative acute kidney injury (AKI) requiring hemodialysis can be regarded as a strong negative prognostic marker for short-term survival. The absence of a clear correlation between most routine RF parameters and survival or the need for early post-operative hemodialysis calls into question the predictive value of pre-operative RF. The negative association of only post-operative hemodialysis on short-term survival emphasises the impact of the occurrence of AKI.

## Introduction

The incidence of heart failure (HF) approaches 10 per 1000 population after 65 years of age^1^. Worldwide, the total number of HF patients exceeds 23 million^2^. Conventional pharmacological and surgical treatment of HF may become unresponsive as the disease progresses^3^. The temporary use of devices for durable mechanical circulatory support (MCS) is an important therapeutic option to avoid the downward spiral of pathophysiological mechanisms consisting of ischemia, hypotension and dysfunction. LVADs use different techniques to relieve left ventricle (LV) function. In the context of an increasing number of patients waiting for heart transplantation as donor heart shortage increases, the importance of LVAD therapy is growing^4^.

The mortality of decompensated HF remains high, even when modern intensive care medicine is initiated early^1^. Short-term outcomes are directly linked to the degree of hemodynamic derangement. Subsequent multiple organ dysfunction is a frequently observed complication of decompensated HF. Cardio renal syndrome (CRS) describes acute or chronic failure of heart or kidney that initiates or perpetuates dysfunction of the other organ, respectively^5^. Renal function (RF) can decline in the context of acute HF due to CRS type 1 (CRS 1)^6^. Chronic kidney disease (CKD), however, often occurs in the context of CRS type 2 (CRS 2)^6^. After LVAD implantation, an initial transient recovery of eGFR is described in literature, but is followed by a late decline of eGFR^7^. Pre-implantation CKD as well as post-implantation acute renal failure are proposed to be a significant predictors of mortality^8–11^. Though an influence on RF has been hypothesised, existing studies do not consider different INTERMACS classes, which support stratification of patients with advanced HF receiving permanent MCS^12^. The present study assessed the influence of LVAD therapy on RF in patients with critical INTERMACS classes as well as the impact of hemodialysis on short-term mortality after LVAD implantation.

## Materials and Methods

This retrospective, single centre observational study evaluates institutional data. Between April 2010 and January 2017, a total of 68 patients with symptomatic end-stage HF were enrolled under the following conditions:

- New York Heart Association (NYHA) classification ≥3
- INTERMACS classification ≤5
- LVAD indication as bridge to transplantation or destination therapy

Before initiating LVAD, cardiologists systematically investigated all patients and conservative heart failure therapy was optimized according European Society of Cardiology guidelines^13^.

Analysis of the patients’ clinical course included full assessment of patient history, laboratory measurements, exertion capacity and need for hemodialysis. The key time-points for data were (i) before LVAD implantation, (ii) immediately following LVAD and (iii) at 30 days post-implantation. eGFR was calculated using the Modification of Diet in Renal Disease (MDRD) equation. Intraoperative data was also recorded. Indication for hemodialysis was confirmed by both, intensivists and nephrologists in every individual case. The study was approved by the ethical committee of the medical faculty of the University Heidelberg (S-663/2017). No specific tissue and/or blood samples were taken at any time deviating from the routine therapy pre-, peri- and postoperatively. According to the ethical approval, no specific individual written consent was necessary for this retrospective analysis of data, which were already present by the routine medical follow up. No experiments on humans were performed, all medication as well as medical devices were completely legally approved by the time of application.

Statistical analysis was processed using the Statistical Package for Social Sciences version 24 (SPSS Inc., Chicago, IL, USA). Variables are given as continuous or categorical variables. Statistical analysis included Kolmogorov-Smirnov test to determine normality of the distribution. Continuous data were shown as mean ± standard deviation and analysed with the Student *t*- test for paired variables. Wilcoxon signed-rank test was utilized for categorical variables. Kaplan-Meier estimator was used to estimate survival function and log-rank test to compare the survival distributions. Cox-regression analysis and binary regression analysis, respectively, was performed for estimating the relationships among variables. Level of significance was *α* = *5%*.

Post-hoc power calculations were conducted using the free available ClinCalc post-hoc power calculator (http://clincalc.com/stats/Power.aspx).

## Results

Patient characteristics at time of LVAD implantation are summarized in Table 1. The study population comprised a cohort of n = 68 patients (82.4% male) with end-stage HF. Their primary cardiac disease was dilative cardiomyopathy (DCMP) (n = 40; 58.8%), of which 6 cases originated from myocarditis, followed by ischemic cardiomyopathy (ICMP) (n = 22, 32.4%) as well as hypertrophic non-obstructive cardiomyopathy (HNCM) (n = 2; 2.94%) and non-compaction cardiomyopathy (NCCM) (n = 1; 1.47%). In 3 cases (4.41%) there was ‘acute cardiac decompensation’ which was not further specified (NFS; n = 3; 4.41%). Pre-operative NYHA functional class was 3.50 ± 0.41.

**Table 1:** Patient characteristics at time of LVAD implantation.

	n	%
**Total**	68	100
**Age (years)**	56.4 ± 9.43	100
**Sex**		
Female	12	17.6
Male	56	82.4
**Heart disease**		
DCMP	40	58.8
ICMP	22	32.4
HNCM	2	2.94
NCCM	1	1.47
NFS	3	4.4
**NYHA functional class**		
I	0	0
II	0	0
III	28	41.2
IV	40	58.8
**eGFR (KDIGO) (ml/min/1.73/m** ^**2**^ **)**		
G1>90	7	10.3
G2 60–89	21	30.9
G3a 45–59	10	14.7
G3b 30–44	16	23.5
G4 15–29	4	5.88
G5 <15	10	14.7

Patient were categorized according to RF (eGFR according KDIGO) prior to LVAD. Normal kidney function (G1) was observed in only 7 (10.3%) patients. Most patients suffered mild to moderate decreased kidney function (G2 = eGFR 60- < 90 ml/min; n = 21, 30.9%, G3a = eGFR 45- < 60 ml/min = 10, 14.7% and G3b = eGFR 30- < 45 ml/min; n = 16, 23.5%). Only 4 (5.88%) patients had severely decreased RF (G4 = eGFR 15- < 30 ml/min). Of those patients with end stage renal failure (G5 n = 10, 14.7%), six (9.82%) needed precursory intermittent hemodialysis. The aetiology of kidney failure was CRS in all patients.

All patients received a continuous-flow 3^rd^ generation LVAD (HVAD Heartware Inc., Framingham, USA) for a severe HF at our institution. Median INTERMACS class was 3.47 ± 1.08. Within the INTERMACS level 1 group (n = 8, 11.8%), 4 patients (50%) had an extra-corporeal life support (ECLS) pre-operatively and 4 (50%) patients received a temporary right ventricular assist device (RVAD) at the time of LVAD implantation. 20 Patients (29.4%) received 22 additional (combined) procedures, of which 7 (31.8%) were tricuspid valve repair, 6 (27.3%) occlusions of a persistent foramen oval/atrial septal defects, 4 (re)-aortic valve replacements (18.2%), 2 (9.1%) atrial ablations, 2 (9.1%) LV thrombus explanations and 1 (4.5%) a surgical left ventricular restoration (4.5%). Over all INTERMACS classes, 11 (16.1%) patients received a temporary RVAD and 1 (1.47%) patient underwent permanent continuous flow BIVAD implantation. Therapy was performed as bridge to transplantation (BTT) in 46 cases (67.6%) or destination therapy (DT; n = 22, 32.4%). 14 (20.6%) patients had a history of previous cardiac surgery.

All implantation procedures were performed under beating heart cardio pulmonary bypass (CPB). During CPB, 38 (55.9%) patients received an intra-operative hemofiltration (Dideco HF 06 Ad, Sorin or Meds HE H5 filter). Right atrial pressure was significantly reduced during operation (15.8 ± 6.4 mmHg to 12.0 ± 3.9 mmHg, *P* = 0.02).

The most common reasons for deaths were cardiogenic shock (n = 5, 35.7%) as well as multiple organ failure (n = 4, 28. 6%), sepsis (n = 2, 14.3% of all deaths) or haemorrhagic complications (neurological n = 2, 14.3% and respectively gastrointestinal n = 1, 7.14%) (Table 2).

**Table 2:** Cause of death 30 days after LVAD (total n = 14).

Cause of death	n	%
Cardiogenic Shock	5	35.7
Multiple Organ Failure	4	28.6
Sepsis	2	14.3
Intracerebral Hemorrhage	2	14.3
Gastrointestinal Hemorrhage	1	7.14

### Significant MDRD improvement after LVAD-implantation

**Figure 1:**
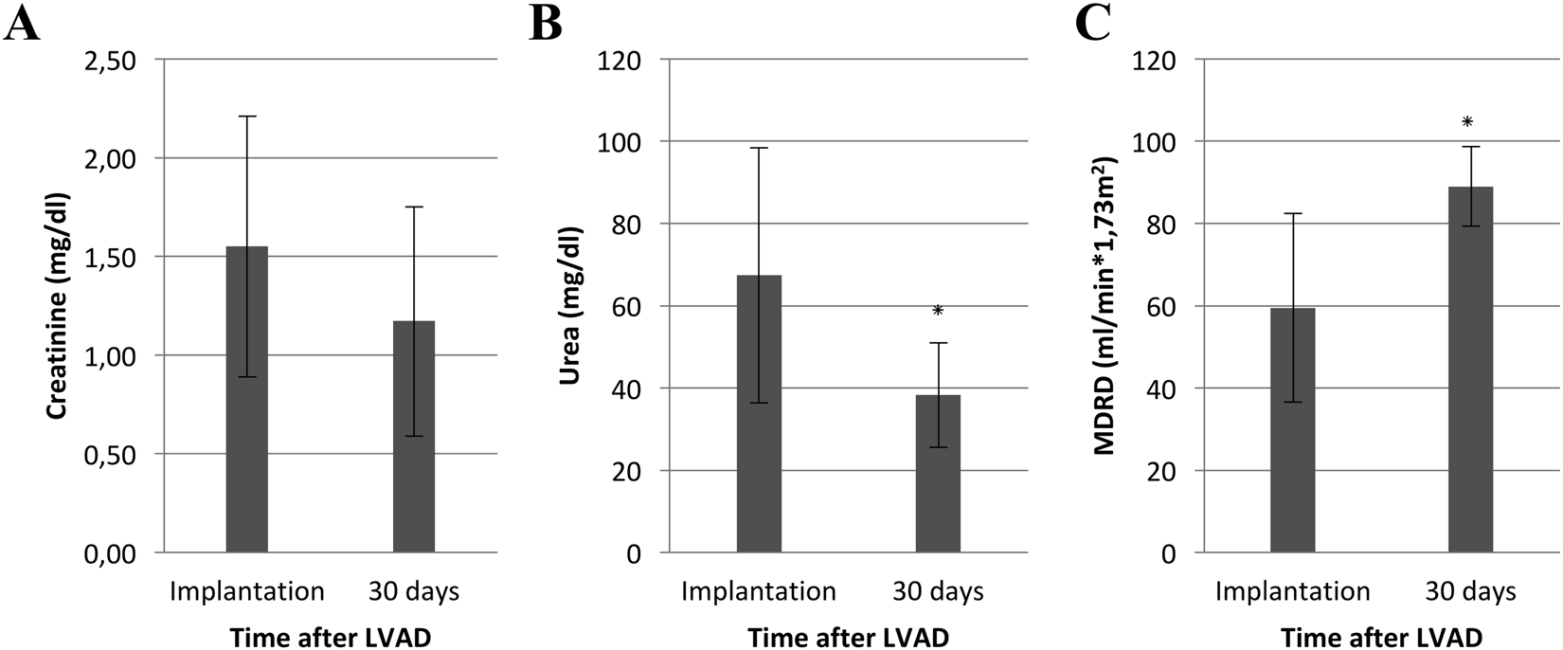
Retention parameters at LVAD implantation and after 30 days. (**A**) Creatinine. (**B**) BUN. (**C**) eGFR ***P < 0.01. MDRD (Modification of Diet in Renal Disease).

30-day follow up was complete in all 68 patients with a mean follow-up time of 27.2 ± 4.46 days. Follow up results of renal variables after LVAD are shown in Fig. 1A–C (not including patients who received hemodialysis at this time point (n = 15, 22.1%). Without hemodialysis, MDRD improved significantly 30 days after LVAD implantation (59.9 ± 24.5 ml/min*1.73 m^2^ to 89.0 ± 22.9 ml/min*1.73 m^2^, *P* < 0.001) (Fig. 1C). Furthermore, blood urea nitrogen (BUN) was significantly reduced 30 days after surgery (68.4 ± 31.1 mg/dl to 38.3 ± 12.7 mg/dl, *P* < 0.001) (Fig. 1B). After 30 days, creatinine levels were reduced without reaching statistical significance (1.56 ± 0.68 mg/dl to 1.17 ± 0.58 mg/dl, *P* < 0.158) (Fig. 1A).

30 days after LVAD implantation, total patient survival was 79.4% (n = 54). NYHA functional class improved from 3.50 ± 0.41 to 2.08 ± 0.58 after discharge from hospital (*P* < 0.001).

### Timing of hemodialysis as risk factor for short-term mortality

Overall distribution of peri-operative hemodialysis is shown in Table 3. Within the 30 day-survival group, 37.0% (n = 20) needed early post-operative hemodialysis, while in the non-survival group (n = 14), 85.7% (n = 12) required early post-operative hemodialysis. At 30 days after LVAD implantation, 15 patients (39.5%) of the survival group were still hemodialysis dependent.

**Table 3:** Distribution of hemodialysis peri-operatively (30 days period).

30 day survival	Hemodialysis					
	pre-operatively		early post-operatively		30-days post-operatively	
	n	%	n	%	n	%
Yes	5	9.26	20	37.0	15	39.5
No	1	7.14	12	85.7	—	—

**Figure 2:**
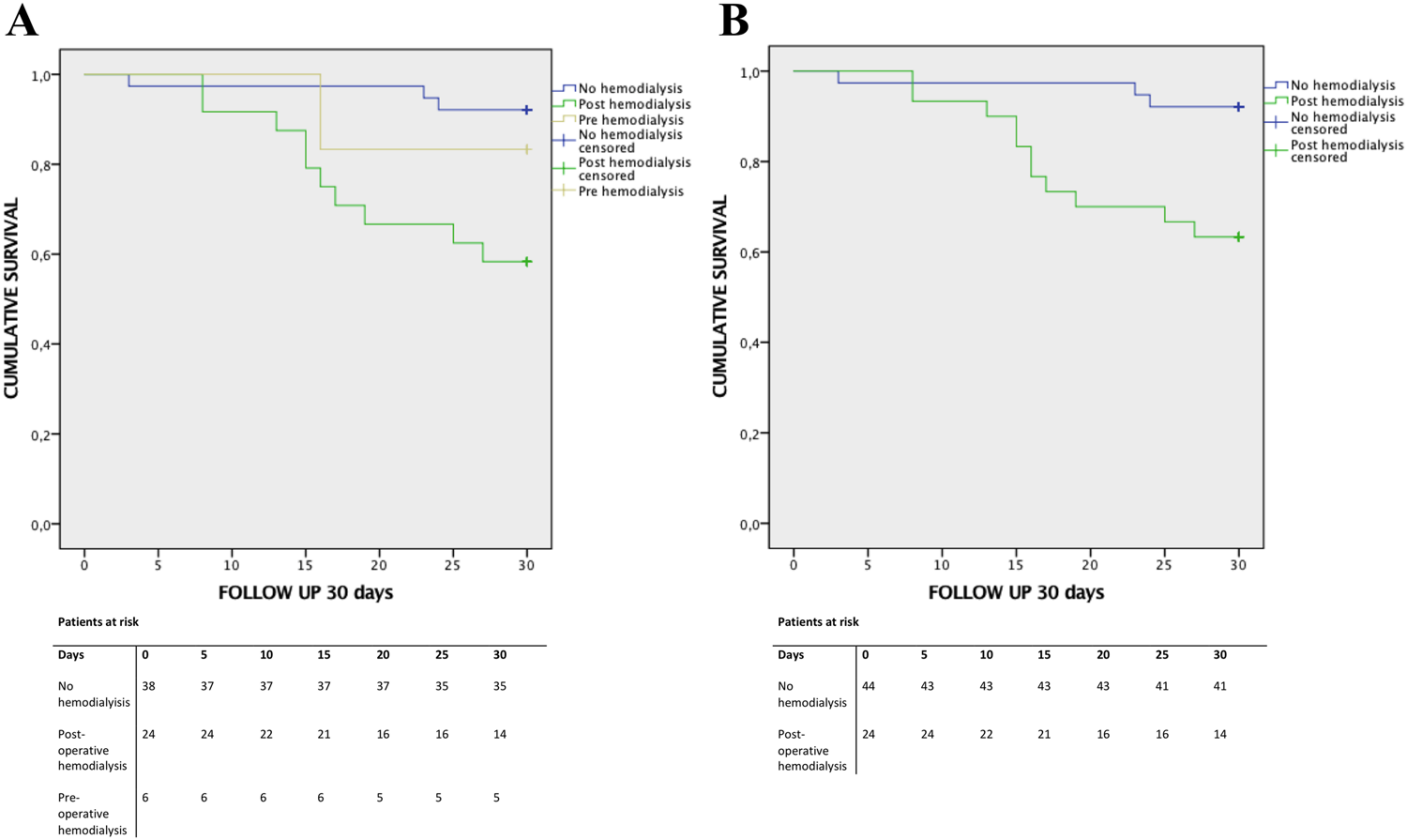
(**A**) Kaplan Meier survival curve 30 days after LVAD implantation. Log rank *P* = 0.004. (**B**) Kaplan Meier survival curve 30 days after LVAD implantation. Log rank *P* = 0.003.

30-day survival was 83.3% (95% CI [23.5, 31.8]) for the group of patients with the need for hemodialysis pre-operatively (n = 6, 8.8%), 58.3% (95% CI [21.2, 27.4]) in the early hemodialysis group (n = 24, 35.3%) and 92.1% (95% CI [27.5, 30.4]) in the non-hemodialysis group (n = 38); (log-rank test *P* = 0.004), as illustrated in Fig. 2A. Of the patients that required hemodylasis pre-operatively (n = 6), one patient (16.7%) had recovery of RF immediately following LVAD. 5 of these patients received early post-operative hemodialysis, but 4 (66.7%) recovered within 30 days (one died within this period; 16.7%). As 5 (83.3%) of pre-hemodialysis patients also received post-operative hemodialysis, another log rank analysis of all post and all not-post-operative-hemodialysis patients was performed (Fig. 2B), revealing a significant higher mortality for patients in need for post-operative hemodialysis (*P* = 0.003).

Cox regression analysis demonstrated that the need for hemodialysis post-operatively decreased short term survival (30 days) significantly (HR 0.171, 95% CI: 0.048;0.613, *P* = 0.002), whereas pre-operative hemodialysis had no impact on mortality (HR 0.585, 95% CI: 0.131;2.617, *P* = 0.511). Concomitant surgery did not influence the likelihood of post-operative need for hemodialysis (HR 1.974, 95% CI: 0.649;6.004, *P* = 0.231).

### Need for RVAD and higher BUN as additional risk factors

Pre-operative levels of creatinine and the eGFR also did not influence mortality (*P* = 0.600 and *P* = 0.222), but higher BUN was associated with increased risk of short-term mortality (HR 1.013, 95% CI: 1.000;1.027, *P* = 0.046). Lower pre-operative albumin levels also did not impact mortality (*P* = 0.569), neither did age (*P* = 0.183), gender (*P* = 0.239) or NYHA classification (*P* = 0.678). Intra-operative change of the right atrial pressure also did not influence mortality (*P* = 0.572). Notably, different INTERMACS classifications did not significantly influence early mortality (*P* = 0.173). Simultaneous RVAD implantation significantly increased short-term mortality (HR 0.062, 95% CI: 0.07;0.544, *P* = 0.012), while overall concomitant heart surgery (*P* = 0.475) or re-operation (*P* = 0.455) had no effect on the mortality. Neither did the general intention of LVAD support (BTT vs. DT; *P* = 0.388).

In a binary logistic regression analysis, the incidence of post-operative hemodialysis was also independent from patient’s INTERMACS classes: Reference category INTERMACS class 5 (*P* = 0.239) did not differ from INTERMACS class 1 (odds ratio [OR] 3.333, 95% CI: 0.557;19.949, *P* = 0.187), likewise INTERMACS class 2 (OR 6.000, 95% CI: 0.873;41.214, *P* = 0.068) as well as INTERMACS class 3 (OR 1.143 95% CI: 0.224;5.841, *P* = 0.873), and INTERMACS class 4 (OR 1.059, 95% CI: 0.276; 4.058, *P* = 0.934) respectively. In the binary logistic regression model of renal retention parameters, only BUN had a significant influence on the probability of post-operative need for hemodialysis (*P* = 0.032, OR 1.018, 95% CI: 1.002;1.036). Neither creatinine (OR 1.469, 95% CI: 0.771;2.799, *P* = 0.243) nor eGFR (OR 1.003, 95% CI: 0.981;1.026, *P* = 0.779) were significant predictors for the need of post-operative hemodialysis. However, lower pre-operative albumin did increase the risk of post-operative hemodialysis (OR 0.874, 95% CI: 0.787;0.969, *P* = 0.011).

## Discussion

End-stage HF is associated with very high one-year mortality^14^, with a one-year survival described as low as 25% in the only medical therapy group^15^. Though heart transplantation remains the gold standard for the definite treatment of severe HF, the shortage of suitable donor organs results in the growing importance for LVAD support as a bridge to heart transplantation strategy^16^. Additionally, in those patients who are unsuitable for a heart transplantation, permanent LVAD is increasingly used as a support for the so called “destination therapy”^17,18^. In fact, destination therapy is growing and accounts for about 38.2% of implants^8^. Emerging research demonstrates the growing potential of LVAD therapy with described benefits even in patients already suffering from severe end-organ dysfunction^19^.

As the number of patients receiving LVAD therapy increases, it is imperative for all specialists involved to further explore the opportunities and benefits of using LVAD as well as better understand the limitations and risks of this therapy.

The annual INTERMACS report states an overall survival in adults with about 80% at 1 year and 70% at 2 years using LVAD with continuous-flow technology (>2,000 implants per year), approaching favourable outcomes after heart transplantation. However, within INTERMACS, up to 30% of LVAD-patients underwent heart transplantation within the first year after LVAD-implantation. Notably, 1-year survival is about 60% in patients still on LVAD support, according to the INTERMACS registry. In addition, Survival is significantly worse using LVAD in lower INTERMACS classes, especially in the short-term (HR 1,69 for INTERMACS 1 and HR 1,44 for INTERMACS 2). Other short-term risk factors identified by the INTERMACS registry are right heart failure (HR 2.57), history of cardiac surgery (HR 1.24) and concomitant cardiac surgery (HR 1.26) as well as unfavourable demographics (older age HR 1.03, female HR 1.32, higher BMI HR 1.10)^8^. Kirklin *et al*. also described the relevance of end-organ function on LVAD outcome. In particular, RF is known to be closely associated with survival after LVAD implantation. The 6^th^ INTERMACS report identified lower albumin levels (HR 0.90, short-term), higher BUN (HR 1.06, short-term and long-term) and higher creatinine (HR 1.05, long-term) as short and long-term negative predictive value for survival. Pre-operative hemodialysis is regarded as strongest negative predictor for survival in the short term (HR 2.37)^8^. However, the results of our study indicate that it is of crucial importance to distinguish between pre- and post-operative hemodialysis, with a strong impact of post-operative hemodialysis on short term mortality. In fact, to the best of our knowledge, the influence of the timing of hemodialysis after LVAD has not been addressed before and requires further investigation.

In the present study, we additionally identified pre-operative hypoalbuminemia and elevated BUN, as clinical markers for a deranged volume status, as independent risk factors for post-operative hemodialysis and in turn, the need for post-operative hemodialysis as a risk factor for short-term mortality following LVAD implantation.

It is difficult to identify authoritative cut-off lab values of renal parameters predicting post-operative kidney function. The MDRD equation tends to underestimate the eGFR for overweight patients and overestimate eGFR for underweight people, since there is no adjustment for body mass.

In the present study, RF monitored by eGFR and BUN significantly improved initially after LVAD implantation. It may be speculated that renal impairment due to CRS 1 (caused by poor renal perfusion) improves with a stabilisation of hemodynamic condition, as stated by Hasin *et al*.^20^. However, creatinine levels decreased post-operatively, failing to reach statistical significance. This might be explained by a better nutrition status and improved muscle mass after LVAD implantation. Nevertheless, in our cohort elevated pre-operative creatinine levels were not associated with a significantly higher mortality. These findings are reflect the results of the annual INTERMACS report, where creatinine was not associated with a higher short-term mortality, whereas BUN did influence short-term outcome^8^. Relevant literature, however, often includes both, serum creatinine levels and BUN, as variables in temporary LVAD risk scores^21,22^. Nevertheless, we could only confirm pre-operative BUN as relevant short-term marker for survival and would encourage re-evaluating about the value of peri-operative retention parameters.

Kirklin *et al*. have described the unfavourable prognostic relevance of renal failure before LVAD implantation^8^. In this study, we could confirm only that post-operative hemodialysis was a negative prognostic marker for short-term survival after LVAD. Conceivably, post-operative renal failure with the need for hemodialysis serves as an early indicator for an insufficient organ perfusion and the subsequent development of multiple organ failure, the timing of hemodialysis seems to be essential to determine prognosis.

Regarding the connection between an unfavourable pre-operative cardiac condition and the need for immediate post-operative hemodialysis, patients were divided into INTERMACS subgroups and analysed according to need for post-operative hemodialysis in a binary logistic regression model. In fact, no statistical significant differences in incidence of post-operative hemodialysis could be demonstrated between critical INTERMACS classifications. Thus, in our patient population, we conclude that poor pre-operative INTERMACS classification does not give any further distinct information about potential renal failure requiring hemodialysis after LVAD implantation. It is of great significance that different low INTERMACS classifications did also not predict short-term mortality sufficiently. It could be suspected, that in critically unwell patients the INTERMACS differentiation might be blurred and INTERMACS classification is only a part of the total clinical picture. Interestingly, concomitant surgery, which is associated with longer procedure time due to the more complex surgery, did not result in more post-operative hemodialysis. Aiming for optimization of cardiovascular status (e.g. by treating intracardiac shunts or valve disease) results in a better hemodynamical status and might preserve kidney function, thus cardio-pulmonary bypass and procedure time (including hypothermic period) might cause damage to the kidneys. A larger study population would be required to explore this further.

Our data suggest that in the case of CRS 1 there is a clear potential for significant recovery of RF according to kidney function parameters after LVAD. This result is in concordance to the literature^23–25^. Within our cohort, even those patients receiving pre-operative hemodialysis recovered within 30 days after LVAD, suggesting better potential for kidney recovery contradicting previous findings^26^. However, within our analysis, pre-operative renal retention parameters such as creatinine and eGFR did not determine the need for hemodialysis in the early post-operative course. In terms of INTERMACS classifications, no difference in renal outcome was monitored between subgroups. In contrast, increased pre-operative BUN and impaired pre-operative albumin are the only predictors for post-operative need for renal replacement therapy. Post-operative hemodialysis, on the other hand, must be regarded as a strong negative predictor for mortality even in the short-term. Kidney function serves as an early indicator for insufficient cardiac output, hence, early detection might prevent early-mortality, especially in patients undergoing LVAD implantation in low INTERMACS levels. As a result, acute post-operative kidney injury is of great prognostic value in the early post-operative period. Notably, none of the patients included in this study suffered from a genuine chronic kidney disease pre-operatively other than CRS. It remains crucial to clearly determine the origin of kidney dysfunction prior to LVAD implantation, as the nature of CKD alters prognosis for recovers sustainably.

There is a strong case to be argued that closer cooperation between cardiac surgeons and cardiologists with specialization in nephrology could help preserve kidney function, particularly as it remains difficult to determine the prognosis of kidney function after LVAD implantation. In addition, a pre-operative and intra-operative approach to optimize volume status is an area that the authors believe must receive increased attention due to its potentially important role in the post-operative course. Most importantly, the diagnosis of pre-operative AKI does not influence outcome after LVAD. Instead, the significant influence of post-operative AKI with subsequent need for hemodialysis should be emphasised. To clearly distinguish the course of AKI and timing of hemodialysis is a key factor to understand the impact of renal function on short-term outcome after LVAD. Additional clinical trials are needed to investigate the impact of different circulatory support strategies on prognosis of end-stage heart failure patients and to help improve short-term and long-term outcomes for these critically ill patients.

## Limitations

The present study reflects results of single-centre retrospective study with a limited number of patients involved. Furthermore, we were not able to conduct power calculations at the beginning of the study to estimate a sample size with sufficient power. However, post-hoc power analysis yielded a power of 0.94, according to our observation of 85.7% hemodialysis requirement early postoperatively in 30-day non-survivors compared with 37.0% postoperative hemodialysis need among 30-day survivors. Therefore, our investigated cohort of 68 patients was sufficient to address our hypotheses. However, our data impose the “real-life” constraint of early LVAD success limited by end-organ failure.
